# Solid mineral potential evaluation using integrated aeromagnetic and aeroradiometric datasets

**DOI:** 10.1038/s41598-024-52270-6

**Published:** 2024-01-18

**Authors:** Arewa James Ogah, Fahad Abubakar

**Affiliations:** 1 ZASTAL, National Space Research and Development Agency, Kano, Nigeria; 2 Department of Geosciences, Confluence University of Science and Technology, Osara, Kogi, Nigeria

**Keywords:** Geomagnetism, Geophysics

## Abstract

The analytical hierarchy process (AHP) was employed to delineate the mineralisation potential across the notable schist belts in northwestern Nigeria. High-resolution aeromagnetic and aeroradiometric datasets were taken into consideration. This was achieved by using advanced signal enhancement techniques to study the structures, identify the hydrothermal alteration zones (that could serve as a pathway for mineralisation), and understand the geologic settings. Amongst the enhancement techniques are first vertical gradient, analytic signal, CET grid analysis and porphyry, Euler deconvolution, and K/Th ratio. The analytic signal reveals lithologic contact, structures and anomalous occurrences that aided the classification of the site into three magneto-lithologic zones: high (> 0.094 nT/m), intermediate (0.028 to 0.094 nT/m), and low magnetic zones (< 0.028 nT/m). The high magnetic zones (HMZ) were considered the main magnetic source outlines, which are inferred to be dominantly intrusive zones for hydrothermal activities. The 3-dimensional Euler deconvolution reveals highly magnetic and intrusive depth sources to be within the range of < 100 to 500 m. The Centre for Exploration Targeting (CET) grid technique revealed the structural distribution from which the lineament density map was produced. The orientations of the prevalent structural anomalies are E-W, NE-SW, WNW-ESE and NW–SE, with similar orientations observed from the first vertical gradient and the analytic signal. The highly dense structural zones coincide with the high magnetic zones and high-frequency amplitudes of the analytic signal and the vertical gradient map, respectively. Additionally, the CET porphyry detects the centres of the intrusive porphyries to be within zones of high lineament density. This reveals that the mineralisation potential of the area is structurally controlled. On the other hand, radioelement maps (eU, eTh, and K%) and ternary maps were used for lithological classification. The radiometric ternary map revealed the highly radioactive zones and the superior concentration of individual radioelements in their respective areas. The K/eTh ratio map delineates highly potassic alteration zones. The AHP model and weighted overlay tool were employed to integrate the analytic signal, lineament density, and K/Th ratio. Consequently, the mineralisation potential of the study site was revealed and classified into high, moderate, and low. This result was validated using known mine sites. There was a total agreement, with 87.5% of mines plotting within the high mineralisation potential class and 12.5% in the moderate class. Promising targets were identified for development.

## Introduction

The availability of raw materials, such as steel, was a major motivator that sparked the industrial revolution of the late eighteenth century. The reason is that most machinery, automobiles, engineering structures, rail lines, etc., are products of ores. Even in this advanced age of industrial development, different nations around the world search for raw materials from the subsurface of the Earth to run machinery, build different types of electric power stations (including nuclear), decorate their environment, and make life more comfortable for their citizens. Any nation must aim for rapid industrial development, invest heavily in iron and steel development, and explore related solid minerals. This explains why many nations worldwide have intensified the search for iron ore, gold, and other solid minerals over the past few decades^1,2^.

Nigeria has depended almost entirely on hydropower for electricity generation since its inception, and recently, other sources like solar, natural gas, and wind turbines have become popular. Nuclear energy, which has become very popular among many developed nations in the world, providing about 10% of global electricity generation, is not yet considered by the country. It is worthy of note that nuclear energy produces zero greenhouse gases, serving as the 2nd largest source of low-carbon energy in the world, ranking next to hydropower. This might be an obvious reason that led countries like France, Ukraine, and Finland to use nuclear energy sources for up to 70%, 51%, and 33% of their total annual electricity output, respectively. Nigeria has the potential to join these nations and many others that use nuclear energy for electric power generation, solid minerals for infrastructural development, and wealth generation. Such a gigantic project can be initiated by searching for the raw materials, that is, by exploring solid minerals and the radioactive potential concealed in some regions of the country^3,4^.

Although combining magnetic and radiometric methods is a powerful tool in exploring solid mineral deposits, in these methods, one does not just rely on direct detection of the orebodies but also on detecting secondary means such as alteration zones or structural features that can serve as favourable areas for the respective mineralisation. In applying the magnetic method, magnetic anomalies are usually produced as indicators of porphyries, kimberlites, alteration zones, massive volcanic sulfides, gold mineralisation, iron ore deposits, etc. Most of such mineralisation occurrences in many regions of the world are known to be structurally controlled^5–12^, so in interpreting the magnetic data, particular attention must be paid to structures, hydrothermal alterations, and intrusive bodies concealed in the magnetic data^13^. This approach has been used in several mineral explorations^14–17^. The radiometric ratios are good indicators for locating alteration zones^18^. Owing to the radiometric survey's capacity to identify possible mineralisations, a number of investigations have been carried out^19–21^.

The search for solid minerals is a global phenomenon, and many researchers have used different approaches and techniques at various locations to achieve their aims. The type of deposits often dictates the kind of geophysical techniques to be deployed; for instance, Guo et al.^13^ discovered that at two different locations in the Gansu Province of China, the ground magnetic method that was deployed could easily detect gold mineralisation associated with host sediments and sulfides, including pyrrhotite. On the other hand, the same magnetic technique failed to detect mineralisation at the third location in the same region, where the response of the igneous host rock prevented the magnetic signal from revealing the mineralisation. Therefore, a researcher must be conversant with the geology of the study area to know precisely the relevant geophysical technique to deploy. Teakle et al.^22^ deployed magnetic and radiometric methods to detect numerous extremely weak magnetic lineaments associated with heavy-mineral sand deposits and outcropping heavy mineral resources rich in thorium in South Australia's Mindarie district. These researchers were able to find numerous new occurrences, including the Halidon deposit, despite magnetic noise brought on by iron oxides in surface sands and some anthropogenic features. Mohamed et al.^1^ also made use of aeromagnetic data, deploying many processing techniques, including analytic signal^23^, first vertical derivative (FVD), total horizontal gradient (THD), tilt angle derivatives^24^, the Centre of Exploration Target (CET) Grid^25^, and the porphyry technique^26^. Other integrated remotely sensed methods have also been prevalent in ore deposit exploration^27,28^. Some other workers concentrated more on delineating regions with high mineralisation potentials, such as hydrothermal alteration zones^29,30^. Lawal et al.^31^ utilised a combination of magnetic and radiometric datasets to delineate mineralisation potential zones in south-western Nigeria, mapping lithological units, structural elements, and hydrothermally altered zones by applying some inversion and filtering techniques such as analytic signals, first vertical derivatives, the Centre of Exploration Target Grid, etc. A more closely related study was that of^32^ which focused on iron mineralisation and tectonic events in the western part of the Zuru schist belt. These workers used aeromagnetic data and geologic field mapping, deploying some inversion and enhancement techniques such as Euler deconvolution, analytic signal, total horizontal derivative, tilt derivative, and reduction to the equator, thereby defining some geologic features, including the boundaries of magnetic units, the geometry of lithologic units, and associated structural features of the area. The workers also mentioned that the fault was a conduit for iron mineralising fluid in the area.

Integrating results from multiple variables in mineral exploration can not be overlooked. The spatial forecast of mineral resources is crucial to effective exploration and resource management. Predictions can be made using a range of factors that affect the presence of minerals in a geologic setting^33^. Therefore, determining the potential for mineralisation is a geospatial problem that can be solved using a variety of factors favourable for mineralisation. Mapping mineralisation potential is frontier work commonly completed during exploration projects to concentrate following exploration efforts on areas that show promise and where spending time and resources will provide the most exceptional results^34^. Critical models such as the analytical hierarchy process (AHP) in the framework of multicriteria decision analysis are appropriate instruments for mineral potential investigation in a frontier geologic setting such as the northwestern Nigerian schist belts. This research aims to map the mineral potential of the northwestern Nigerian schist belt in an unbiased and reliable manner, taking AHP into account. The study intends to lower systematic ambiguities and improve understanding of the underlying geological control of mineralisation by contrasting and evaluating the significance of each exploration criterion. Employing this approach is crucial to integrating aeromagnetic and aeroradiometric analyses in northwestern Nigeria, where the potential for mineralisation is uncertain.

Several related research studies have been conducted in northwestern Nigeria (study area inclusive) for its mineralisation potential. However, most of this research was conducted in pockets of the study area; for example, Andongma et al.^35^ mapped the alteration zones favourable for gold mineralisation within the Malumfashi schist belt. Arogundade et al.^19^ analysed the mineralisation potential of some parts of Zamfara, Nigerian sheet 75 (on a scale of 1:100,000) using integrated geophysical datasets. Augie et al.^36^ evaluated the gold mineralisation potential of some parts of the study area using magnetic datasets. Salawu et al.^37^ utilised the remote sensing and geophysical datasets to delineate structures favourable for gold mineralisation in the southern axis of the Zuru schist belt. Some other geochemical studies have also been carried out within the study area (including^38,39^). However, the present study assessed the mineralisation potential of northwestern Nigeria (covering Nigeria sheets 73, 74, 75, 76, 77, 96, 97, 98, 99, 100, 118, 119, 120, 121, and 122 on a scale of 1:100, 000) using AHP systems and weighted overlay tools. Aeromagnetic and aeroradiometric data analysis were taken into account in the assessment. The work aims to develop exploration techniques using a multicriteria approach to evaluate the structures and hydrothermal alteration zones favourable for mineralisation across the notable schist belts. The results of this assessment of the potential mineralisation may stimulate interest in exploration and exploitation in Nigeria.

### Description of the site and its mineralisation potential

**Figure 1:**
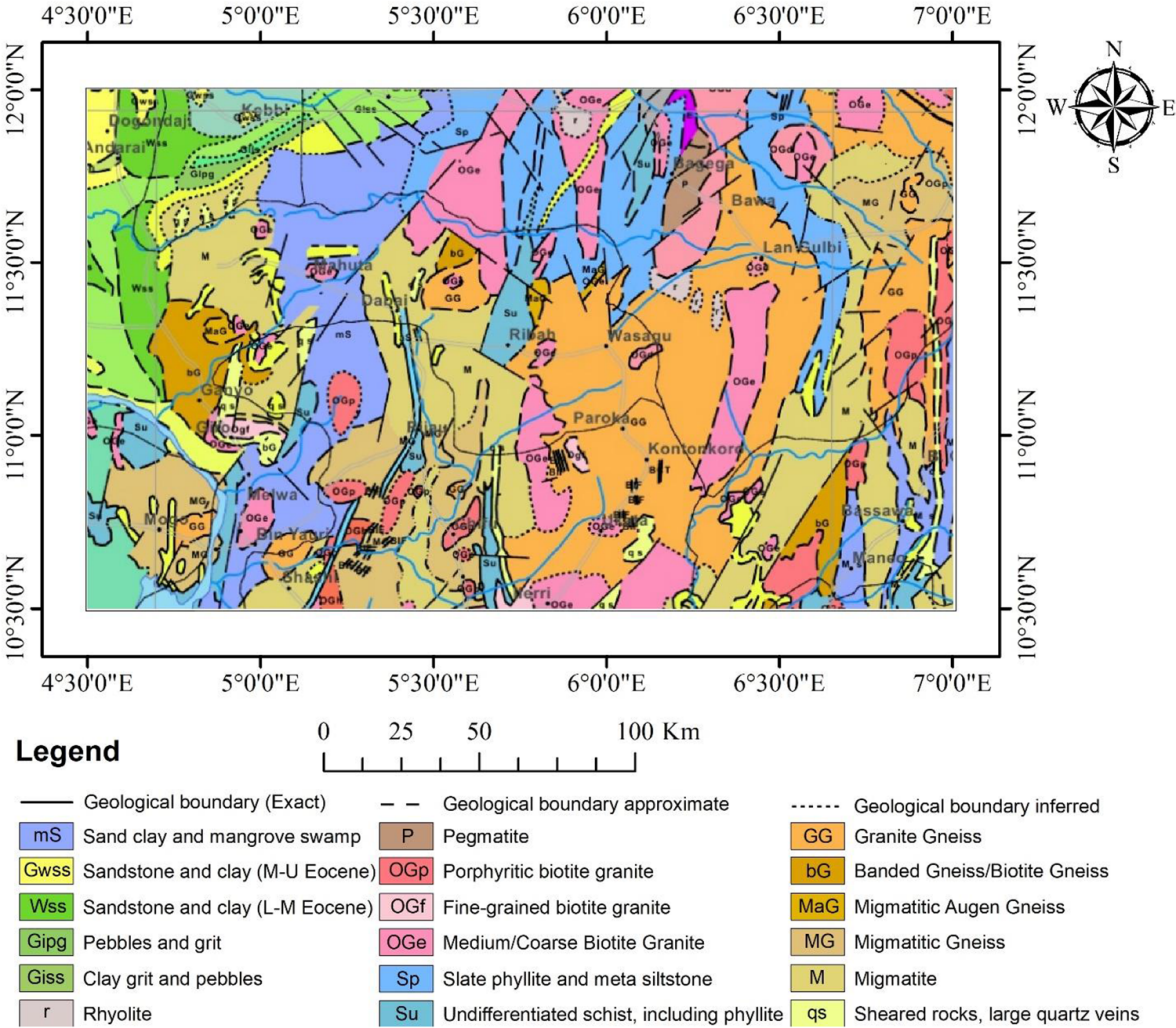
Map showing the geological distribution of the site.

The research area is in West Africa, and it falls entirely within Nigeria (Fig. 1). It covers some parts of Zamfara, Kebbi, Katsina, Kaduna, and Niger State. The area of the site covered is 45,991 km^2^. It is the eastern part of the West African Craton^40^. It has been reported to host a variety of mineral resources, including iron ores, gold, etc.^41^. The area is covered by migmatitic Precambrian basement rocks, Proterozoic metasedimentary (schist) belts, granitoids, and Tertiary sediments.

The migmatitic Precambrian basement rocks within the site consist of sheared rocks, migmatite, migmatitic gneiss, banded gneiss, and granite gneiss. The Proterozoic metasedimentary belts are made up of schist (undifferentiated), phyllite, and slate. These schist belts captured within the area of study are the major schist belts in Northwestern Nigeria. It comprises the Zuru, Anka, Kushaka, Ushama, Kusheriki, and Birni Gwari schist belts. Other schist belts like Maru and Wonaka also fall within the study area^42^. The granitoids comprise fine-grained, coarse-grained, porphyritic granites and pegmatites of Pan-African age^3^. The Tertiary sediments are those of the Sokoto Basin, which consist of sandstones, siltstones, and claystones^3^.

The metasedimentary belts of the Precambrian age (study area inclusive) host very important economic deposits such as the Banded Iron Formation, gold, and marble^43^. Geologic structures usually control these deposits and are often associated with hydrothermal alteration^37,44^. The geologic structures serve as conduits for the mineralisation and, of course, enhance the migration of hydrothermal mineralising fluid^44^. The schist belts within the area exhibit similar geologic settings. Banded iron formation (BIF) has been reported to be associated with many of the Northwestern schist belts, including Maru, Muro, Birnin-Gwari, Kushaka, Kazaure, Malumfashi schist belts, etc.^45^. Gold occurrences have also been reported to be associated with the schist belts^3,46,47^.

### Data and methodologies

The high-resolution aero-radiometric and aero-magnetic data of Nigerian sheets 73, 74, 75, 76, 77, 96, 97, 98, 99, 100, 118, 119, 120, 121, and 122 (on the scale of 1:100, 000) was obtained from the Nigeria Geological Survey Agency, Abuja, Nigeria (NGSA). Table 1 contains the essential acquisition parameters. However, other details can be found on the NGSA website (ngsa.gov.ng). Both data were analysed using Geosoft (Oasis Montaj) version 8.5, ArcGis 10.5, and Rockwocks 17.

**Table 1:** Acquisition parameters for the aero-radiometric and aero-magnetic survey.

Parameter	Survey specification
Mean sensor ground clearance	80 m
Flight line separation	0.5 km
Interval of tie line	5 km
Recording interval	0.1 s

### Aeromagnetic data

**Figure 2:**
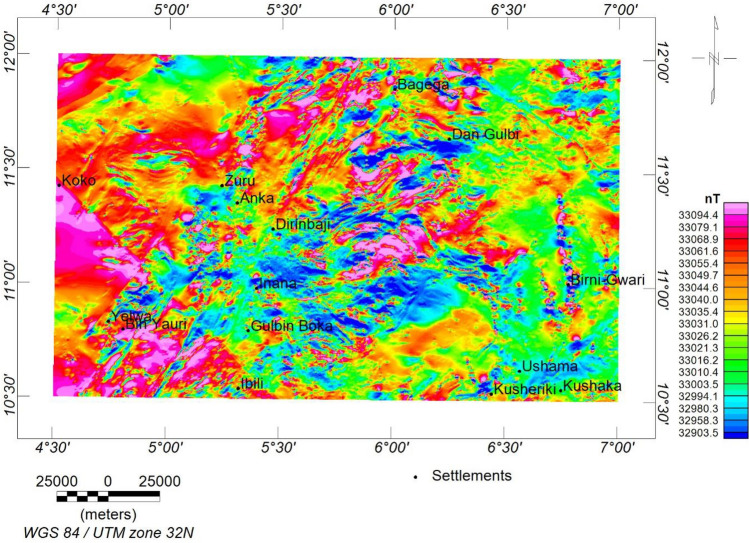
TMI map of the research site.

**Figure 3:**
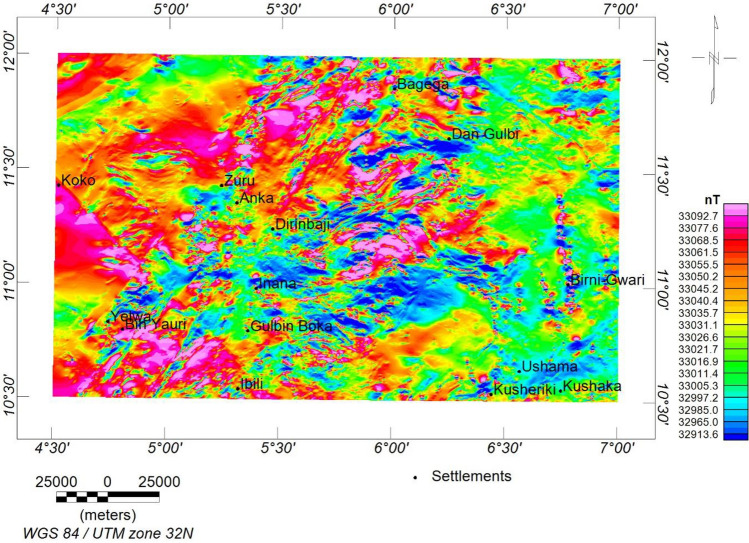
RTE map of the research site.

**Figure 4:**
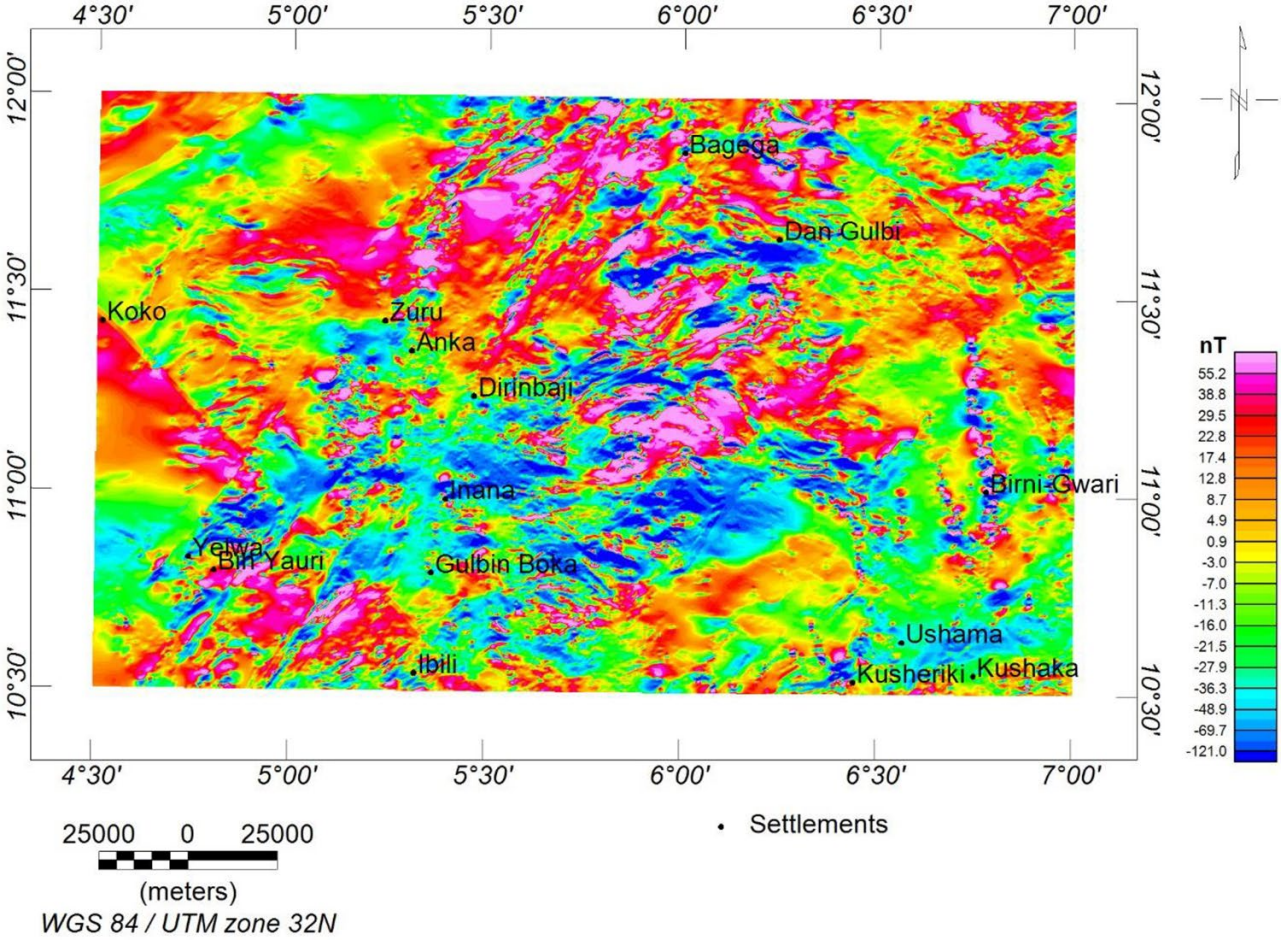
Residual map of the research site.

The data was in the form of a total magnetic intensity (TMI) grid (Fig. 2). The magnetic inclination (− 0.36) and declination (− 1.83) were obtained based on the International Geomagnetic Reference Field (IGRF) 2005 on the magnetic calculator to generate the reduction to the magnetic equator (RTE) map (Fig. 3). The RTE transformation centres the peaks of the magnetic anomalies over their causative magnetic sources, eliminating asymmetry related to low latitude magnetic anomalies and the impact of magnetic inclination^48,49^. Furthermore, the regional-residual separation was performed to obtain the residual grid using the least squares polynomial fitting technique^50,51^. The theoretical background of the method can be found in^50–53^. The residual grid (Fig. 4) was further subjected to several filtering techniques to enhance the qualitative and quantitative interpretation signal. Table 2 has a summary of the filtering techniques employed in this study.

**Table 2:** Signal enhancement technique employed for the aero-magnetic data.

Technique	Explanation	Its application
Reduction to the Equator	It is an algorithm that accounts for a spatial shift, especially in an equatorial zone^54,55^	It is used to place the magnetisation of a geologic body over its causative sources^55,56^
Regional-residual separation	It is the most efficient procedure to obtain residual field^57^. The residual field is the deviation from the regional field and is considered the target source susceptibility^50^	To reveal good target source susceptibility for exploration^58,59^
Vertical gradient	It is defined as the gradient of magnetic intensity^33^ given as: $\frac{\partial MI}{\partial z} (1)$, where $MI$ is the magnetic intensity	To enhance and highlight shallow features and sharpen the edges of anomalous bodies^60^
Analytic Signal (AS)	According to^23^ AS is given as: $\sqrt{{\left(\frac{\partial MI}{\partial x}\right)}^{2}+{\left(\frac{\partial MI}{\partial y}\right)}^{2}+{\left(\frac{\partial MI}{\partial z}\right)}^{2}} (2)$ Where $\frac{\partial MI}{\partial x}$, $\frac{\partial MI}{\partial y}, {\text{and}} \frac{\partial MI}{\partial z}$ are gradients in the x, y, and z directions, respectively	To define geologic borders and resolve and highlight anomalous sources that are closely spaced (especially in equatorial regions)^61^
Euler deconvolution	It is an algorithm that rapidly assesses a huge magnetic dataset, utilising a gradient of anonymous source geometry^33,62^	To estimate the depth of magnetic sources^63^
CET grid analysis	It is a tool developed by geophysical explorers for magnetic structural analysis^25,64,65^	To reveal magnetic lineaments^66^. This was further used to generate the lineament density map in this study
CET porphyry analysis	Like the CET grid analysis, this has to do with porphyry detection^26^	To reveal intrusive porphyry centres^67^

### Aeroradiometric data

The radiometric survey measures the naturally occurring radioactivity of gamma rays. This gamma radiation emanates from minerals having radioactive isotopes of uranium (U), thorium (Th), and potassium (K). Although there are more than 50 naturally occurring radioactive elements, just three—K, U, and Th—emit most terrestrial radiation. Among the geophysical techniques, the radiometric method stands out in some ways. Firstly, it has only a minimal ability to probe into the subsurface because it analyses radioactivity that only originates from the shallow portion of the Earth's crust. Second, radiometric data are used to study variations in the chemical makeup rather than the physical properties of the survey region since it is feasible to determine the elemental source of the radiation from the energy of the emitted rays. The analysis of radiometric data straddles the geochemistry-geophysics divide^33^. Due to the differing radioactive fingerprints, the high-resolution aeroradiometric survey is capable of mapping lithologies, detecting zones of hydrothermal alterations, deciphering highly radioactive geologic deposits, revealing surficial structures, and determining the concentration of the radioelements (eU, eTh, and K)^33,68^.

An essential component of the mineralisation interpretation of radiometric data is the integration of the three radio elemental concentration channels, K, eU, and eTh, with the TC channel and other relevant data. The abundance of the radioelements is useful in locating possible host rocks favourable for mineralisation^69^. However, the abundance ratios eU/eTh, eU/K, and eTh/K are frequently more diagnostic of lithology and hydrothermal alteration changes than the abundances of the individual radioelements^70^. However, eU/K, eU/eTh, and eU are very important for delineating uranium deposits^71^, K/eTh for delineating hydrothermal alterations^27^ and potassic hydrothermally altered zones^18^. This study generated maps to identify lithological units by analysing K, eU, and eTh radioelements in conjunction with the ternary images, and the K/eTh ratio was used to delineate the hydrothermal alteration zones in the survey area.

### Spatial integration and correlation

#### Analytical hierarchy process (AHP)

AHP is an extremely flexible resource allocation, prediction, dispute resolution, and multicriteria decision-making technique. Ratio scales are created by converting continuous or discrete paired comparisons. These comparisons (as a matrix) can be performed using a simple scale that indicates the degree of predisposition^72^. The comparison matrix's mathematical background is available in^73^. AHP places a high value on measurement variations, relationships within and across structural piece groupings, and consistency deviations^73^. In order to compare the criteria on a scale of 1 to 9 (Table 3), critical viewpoints from experts were used in this study. The AHP model was then used to assign appropriate weight values^72,74–76^.

**Table 3:** Basic AHP scoring scale.

Score	Significance
1	Two equally important variable
3	A little more significant
5	Much more significant
7	Strongly more significant
9	Extremely more significant
2, 4, 6, 8	Midway values between the two neighbouring judgments
½, 1/3, …, 1/9	These are the significance levels' reciprocals

A consistency check must be performed since priority may only be generated from consistent or substantially constituent matrices. In relation to the eigenvalue approach, a consistency index (CI) was introduced by^77^, and it is given as follows:

3 $$CI = \frac{{\lambda_{large} - d}}{d - 1}$$

where ${\lambda }_{large}$ is the largest eigenvalue in the pairwise comparison matrix, and $d$ represents the matrix's dimension.

As stated in^72^, a study's conclusion and judgment are considered valid if the consistency ratio (CR), expressed as follows, is less than 0.10. CR is given as:

4 $$CR = \frac{CI}{{RI}}$$

where RI is the mean random consistency index.

#### Factors considered and reclassification in the AHP analysis

Lineament density, analytic signal, and K/eTh ratio were considered for the mineralisation potential assessment. The lineament density shows an area's structural concentration distribution. These structures may serve as conduits for mineralisation. Most minerals and potential mineralisation within and beyond the study area have been attributed to geologic structures and hydrothermal alterations^8,10,35,78–80^. Lineament density has been used as a key parameter in several mineral explorations^80–83^. The analytic signal resolves and highlights anomalous magnetic sources that are closely spaced. It can reveal magnetic contacts and structures^84^ and is suitable for analysing magnetic data in an equatorial region like Nigeria^61,85^. It has been used as a veritable tool for several mineral exploration purposes^5,86,87^. The K/eTh ratio is very effective in delineating hydrothermal alteration zones^33^. A lot of research has been done using the K/eTh ratio for mineralisation mapping^20,70,79,88,89^.

These parameters designated for the mineralisation potential were processed as raster layers. To perform reclassification, the features that represented the rasters were reclassified using units of mineralisation favorability. The classification was made and the standard deviation of individual parameters was also considered. This was done in the ArcGIS (10.5 software) environment. This was further processed using weighted overlay tool.

#### Weighted overlay

By superimposing many raster layers and assigning weights to each layer according to their importance, the weighted overlay method can be used to build maps^81,90^. This technique was employed to integrate the contributing parameters in the prospect for mineralisation potential. According to their influence, AHP was used to assign the parameters for the class range, suitability score, suitability degree, and the weight (%) of the thematic layers (Table 4). The mineralisation potential map was produced using the software's weighted overlay tool.

**Table 4:** Parameters employed in the weighted overlay analysis for the mineralisation potential of the study area.

Raster layer	Range of class	Suitability score	Suitability degree	Weight (%) assigned
Lineament density (m/km^2^)	> 0.125 0.050 – 0.125 < 0.050	1 2 3	High Moderate Low	33
K/eTh (%/ppm)	> 0.142 0.084 – 0.142 < 0.084	1 2 3	High Moderate Low	26
Analytic signal (nT/m)	> 0.094 0.028 – 0.094 < 0.028	1 2 3	High Moderate Low	41

## Result and discussion

### Aeromagnetic results

#### First vertical gradient map (FVGM)

**Figure 5:**
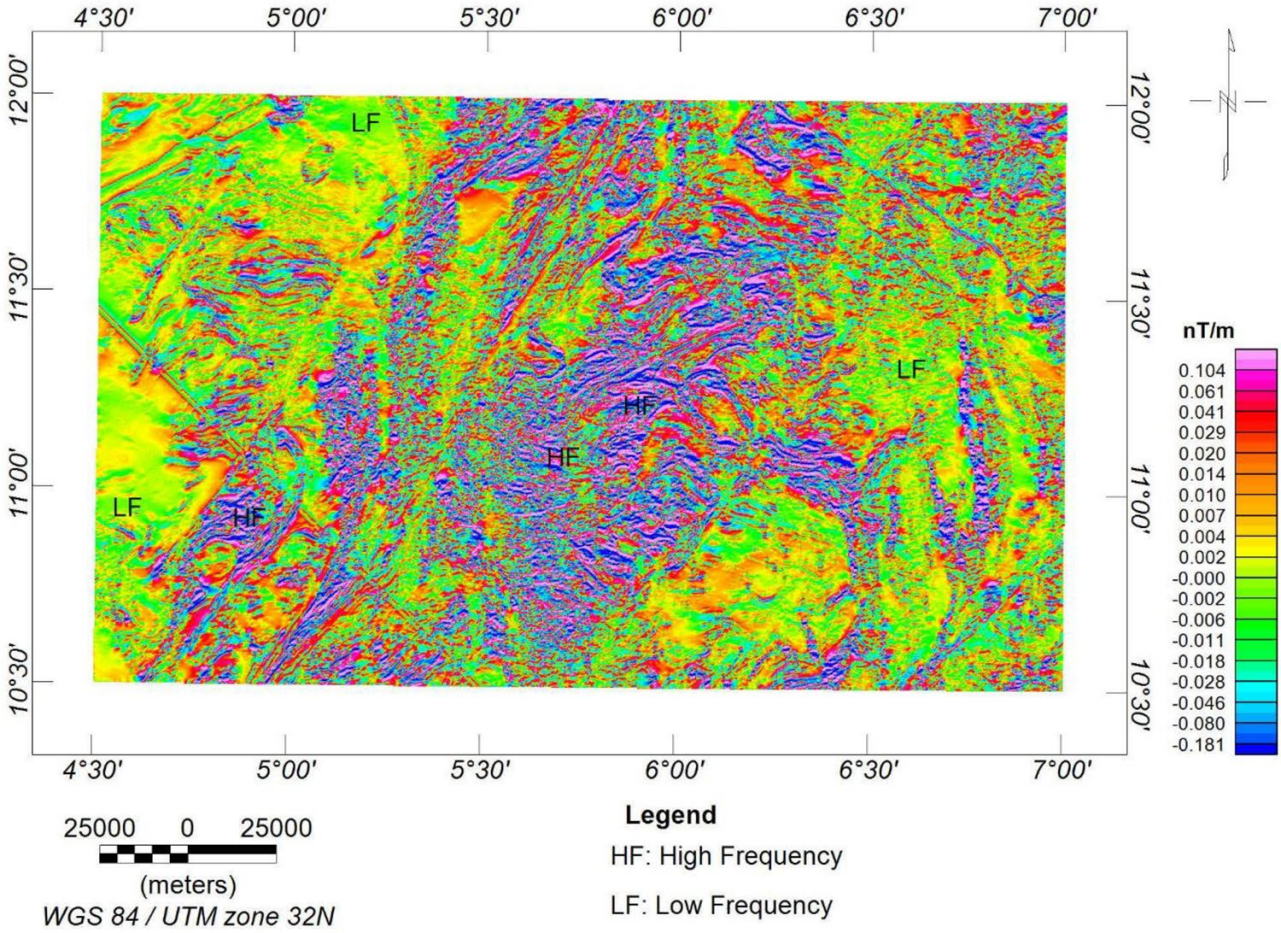
First vertical gradient map.

**Figure 6:**
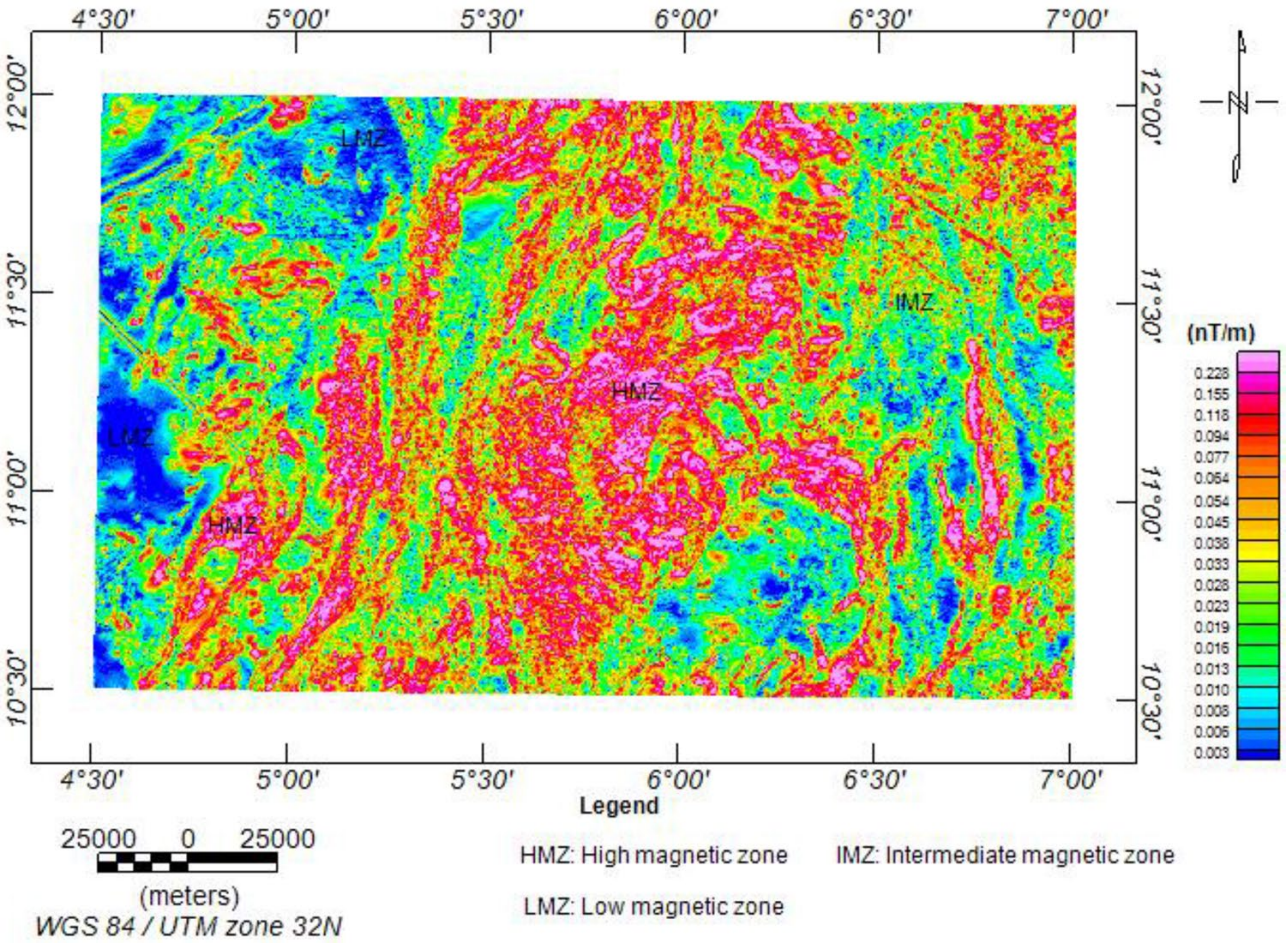
Analytic signal map.

The FVGM reveals local structural anomalies with orientations of E-W, NE-SW, WNW-ESE, and NW–SE (Fig. 5). NE-SW orientation was obtained by^36^. The amplification of these structural characteristics (faults, fractures, and folds) notably reflects structures that could serve as a main conduit for hydrothermal activity^6,7^. It is interesting to notice that short-wavelength anomalies were concentrated in the research area's central, northcentral, and southcentral portions, indicating a shallower depth of the causal sources compared to the other regions, where deeper source inferences can be made. The analytic signal map (Fig. 6) and the FVGM exhibit very similar signatures, such that the vertical gradient's high and low amplitude frequencies are consistent with the analytic signal's high and low magnetic zones, respectively. This high-amplitude signal could result from magmatic injections, resulting in granitic and metasedimentary rock assemblages. Identical signatures were also observed from the CET grid analysis.

#### Analytic signal map (ASM)

The ASM shows all the edges of anomalous occurrences, structural patterns, and lithological contacts dependent on the magnetisation of different rock compositions^80,89^ (Fig. 6). This made it possible to categorise the entire study area into three main magneto-lithologic zones: high (> 0.094 nT/m), intermediate (0.028 to 0.094 nT/m), and low magnetic zones (< 0.028 nT/m). The high magnetic zones (HMZ) can be referred to as the main magnetic source outline, which is inferred to be dominantly intrusive zones, resulting in (a) the formation of granitic rocks (including pegmatites, porphyritic granite, and biotite granite) and (b) the metamorphism of preexisting rocks into undifferentiated schists, granite gneiss, biotite gneiss, migmatite, and migmatite gneiss. The intermediate zones (IMZ) correspond to the younger metasediments, such as the phyllites, while the low magnetic zones (LMZ) correspond to the sedimentary terrains consisting of clay, pebbles, and sandstones. Augie et al.^36^ obtained 0.001 to 0.005 nT/m, 0.006 to 0.0043 nT/m, and > 0.048 for low, moderate, and high magnetic zones, respectively, in the western half of the study area.

#### 3-dimensional euler deconvolution (3-DEuD)

**Figure 7:**
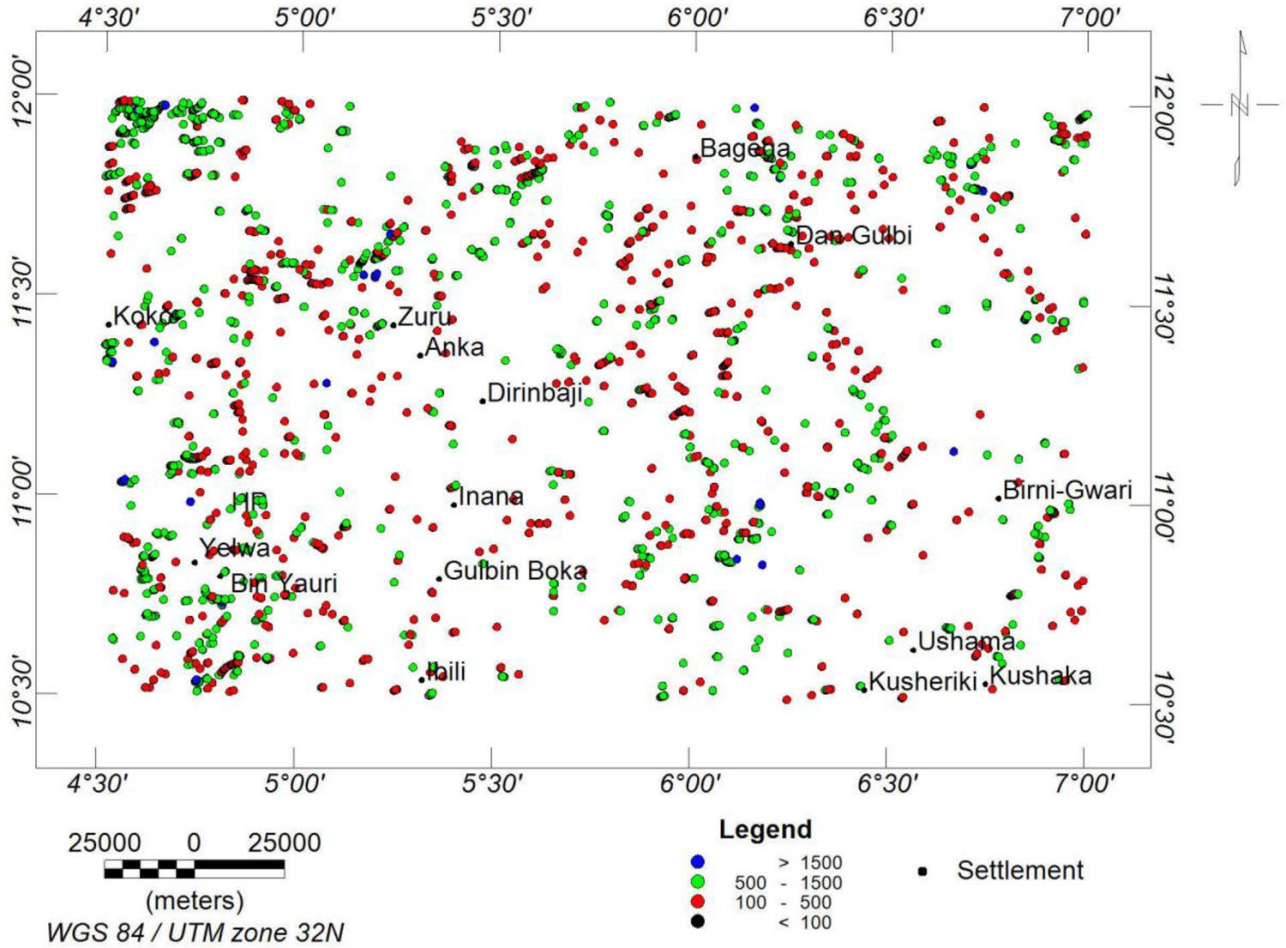
3-DEuD depth solutions.

The 3-DEuD was used to pinpoint the positions and depths of anomalous magnetic sources. This was achieved by defining the source body's suitable structural index (SI). For a source with a specific geometry, the structural index is an exponential factor representing the rate at which the field diminishes with distance. In the case of the investigation at hand, intrusive structures are of paramount importance. Therefore, the concordant (sill) and discordant (dyke) intrusive geological models were employed, which implies a SI of 1^62,91^. The result of the Euler depth shows the depth of magnetic sources range to be < 1500 m, 500 to 1500 m, 100 to 500 m, and < 100 m. Most of the highly magnetic structures and intrusive depth sources are within the range of < 100 to 500 m (Fig. 7). The depth estimate of mineralisation potential sources in the western axis of the study area was estimated to be between 250 and 375 m^79^. The results of the Euler deconvolution compared with the ASM, FVGM, and lineament density map indicate that the shallow depth range (< 100 to 500 m) corresponds to the high magnetic zone, high frequency, and high lineament density, respectively.

#### CET grid and porphyry analysis

**Figure 8:**
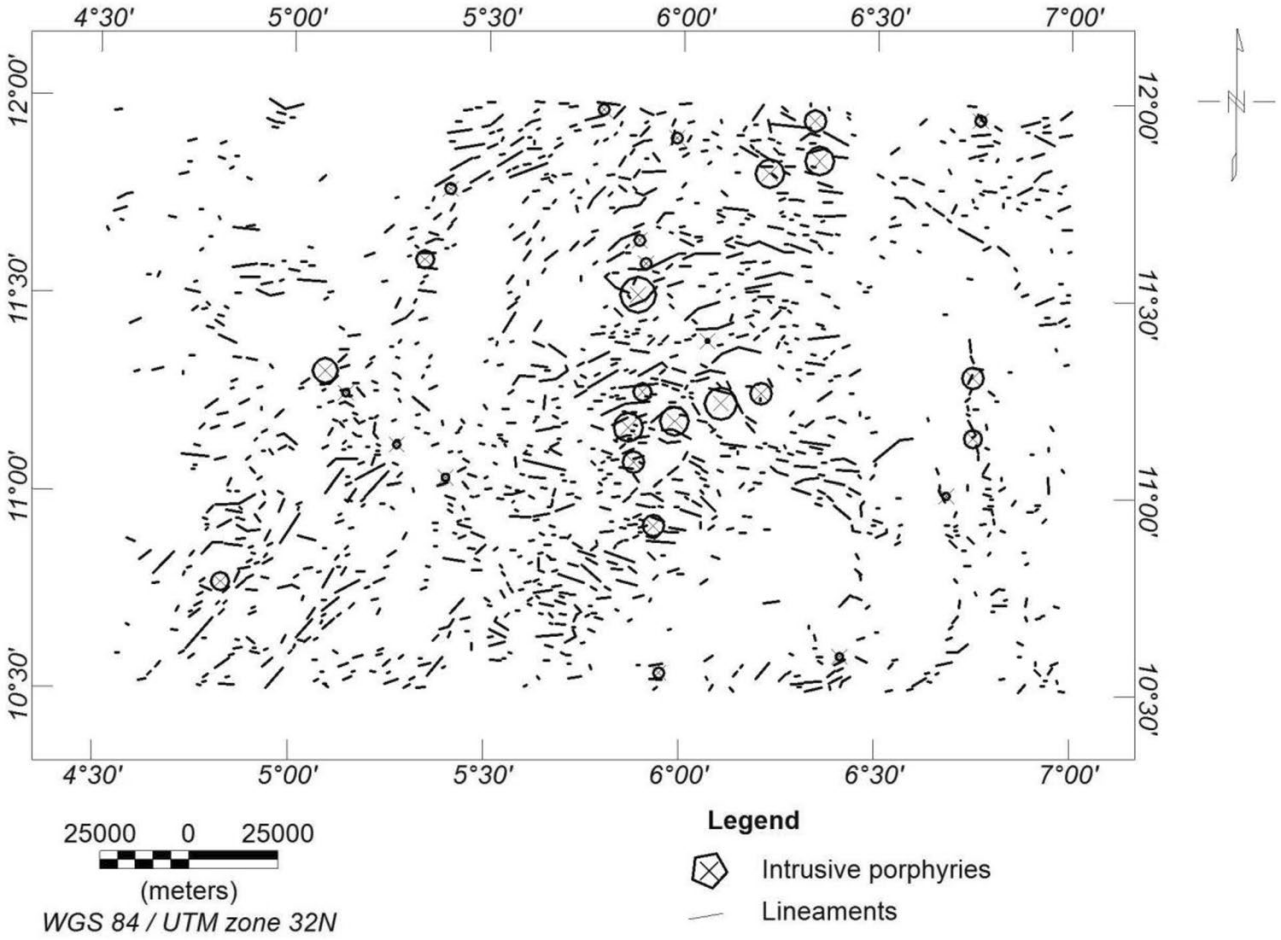
CET Grid and Porphyry map of the research site.

**Figure 9:**
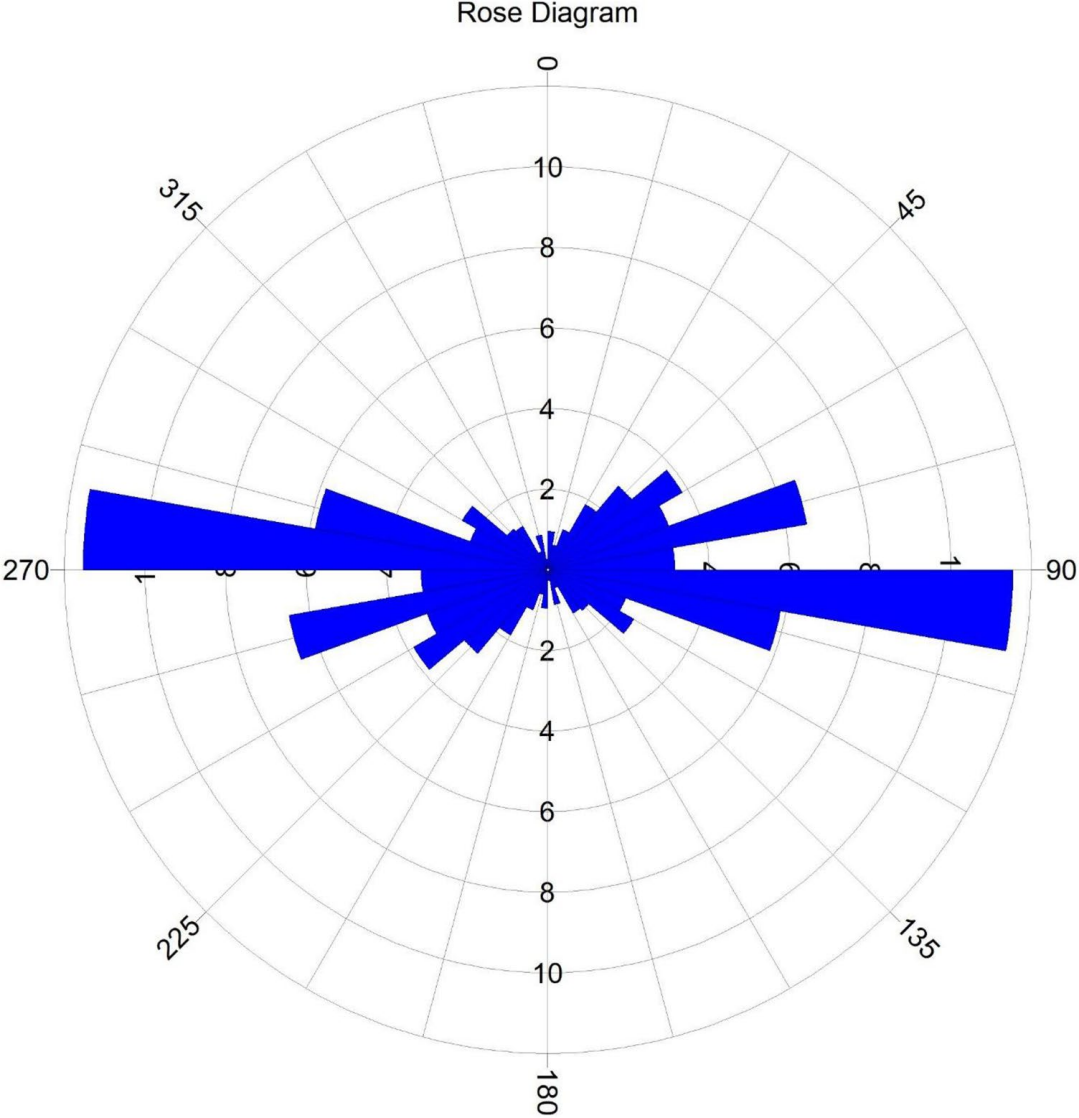
Rossete diagram showing the directional distribution of structural trend within the research site.

**Figure 10:**
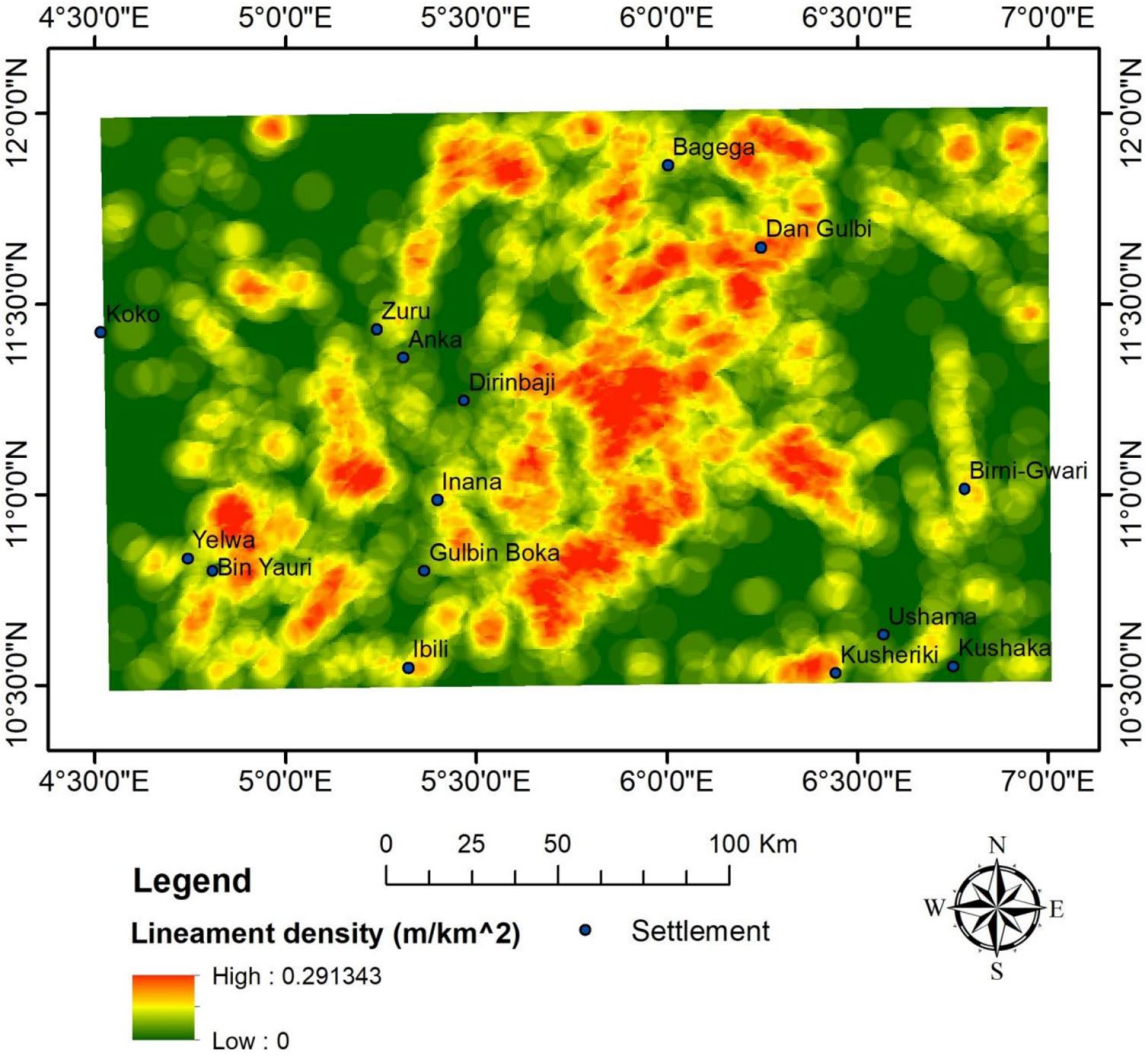
Lineament density map of the study area.

The CET grid technique was used to extract the structures (i.e., lineaments) within the research site (Fig. 8). The dominant structural trends are E-W, NE-SW and WNW-ESE (Fig. 9). A similar structural trend was obtained by^19^. The areas with a very high density of lineaments coincide with the high magnetic zones and high-frequency amplitudes of the ASM and the FVGM, respectively. The mineralisation potential of the area appears to be structurally controlled. The structures are responsible for the hydrothermal mobilisation and concentration of mineralising fluids. The CET porphyry is used to detect the centres of potential mineralisation zones, which are exploration targets^25,92^. These porphyries were plotted on the lineament map (Fig. 8) and fall within zones of high lineament density. However, to reveal the mineralisation potential zones of the site, a lineament density map was produced from the lineaments generated (Fig. 10).

Lineaments have long been thought to interact significantly with mineral deposits by offering pathways for mineralised fluids to concentrate into the upper crust. As a result, it has been actively used as a reference in mineral exploration^25,80^. Therefore, the zones of high lineament density are the areas with high mineralisation potential^28,80,83^.

### Aeroradiometric result

#### eU, eTh, and K% concentration maps

**Figure 11:**
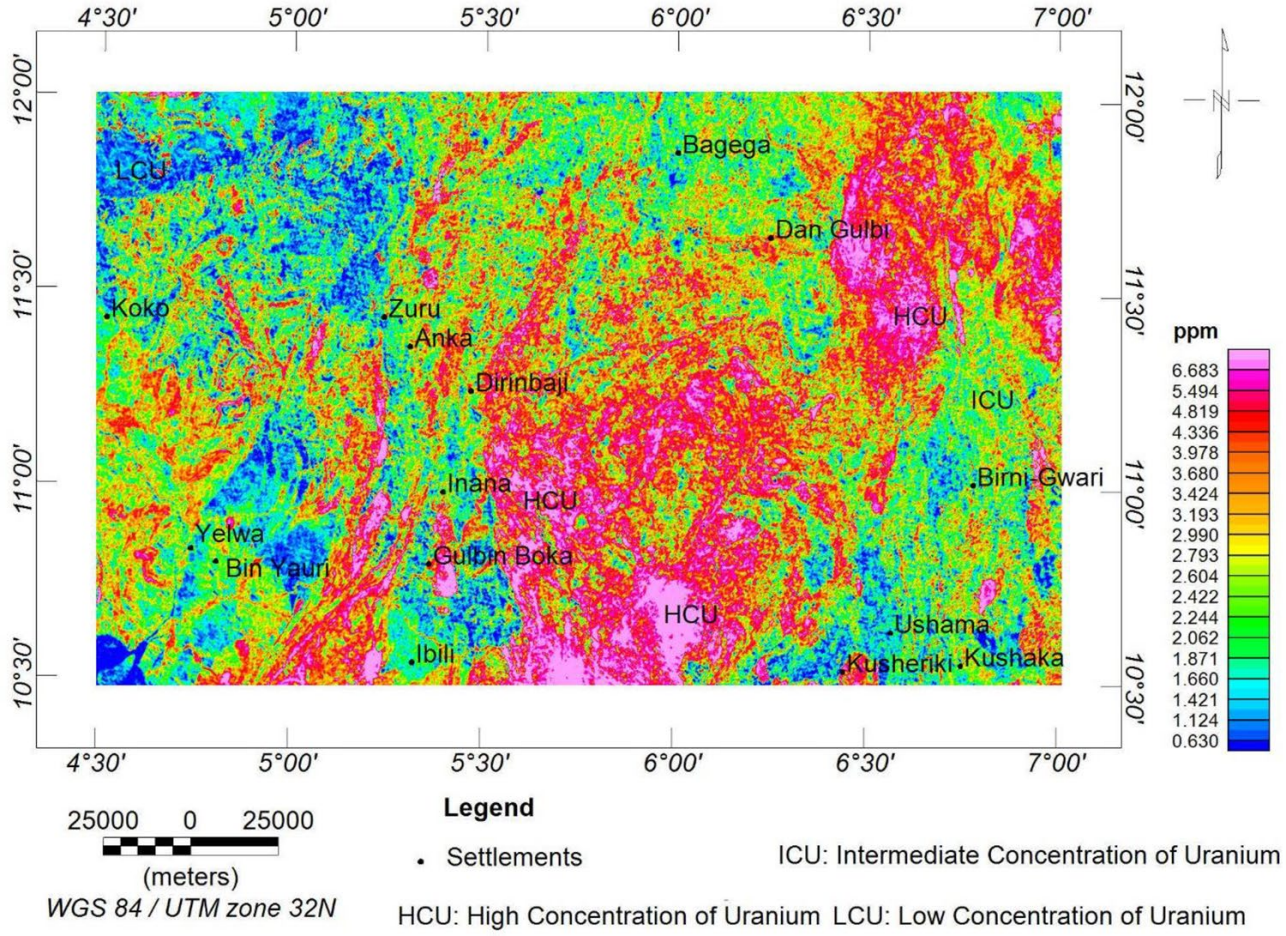
eU concentration map of the research site.

The equivalent concentration of uranium (eU) map (Fig. 11) reveals a high-level concentration of uranium to be between 3.7 and > 6.7 ppm. This range of concentration is associated with porphyritic granite, biotite granite, granite gneiss, and migmatite rocks. The intermediate concentration range between 2.2 and 3.7 ppm represents the metasediments. This is simply because uranium concentration is not significantly affected during metamorphism^93^. At the same time, low concentrations between 0.6 and 2.2 ppm correspond to the clay, sandstones, pebbles, and grits.

**Figure 12:**
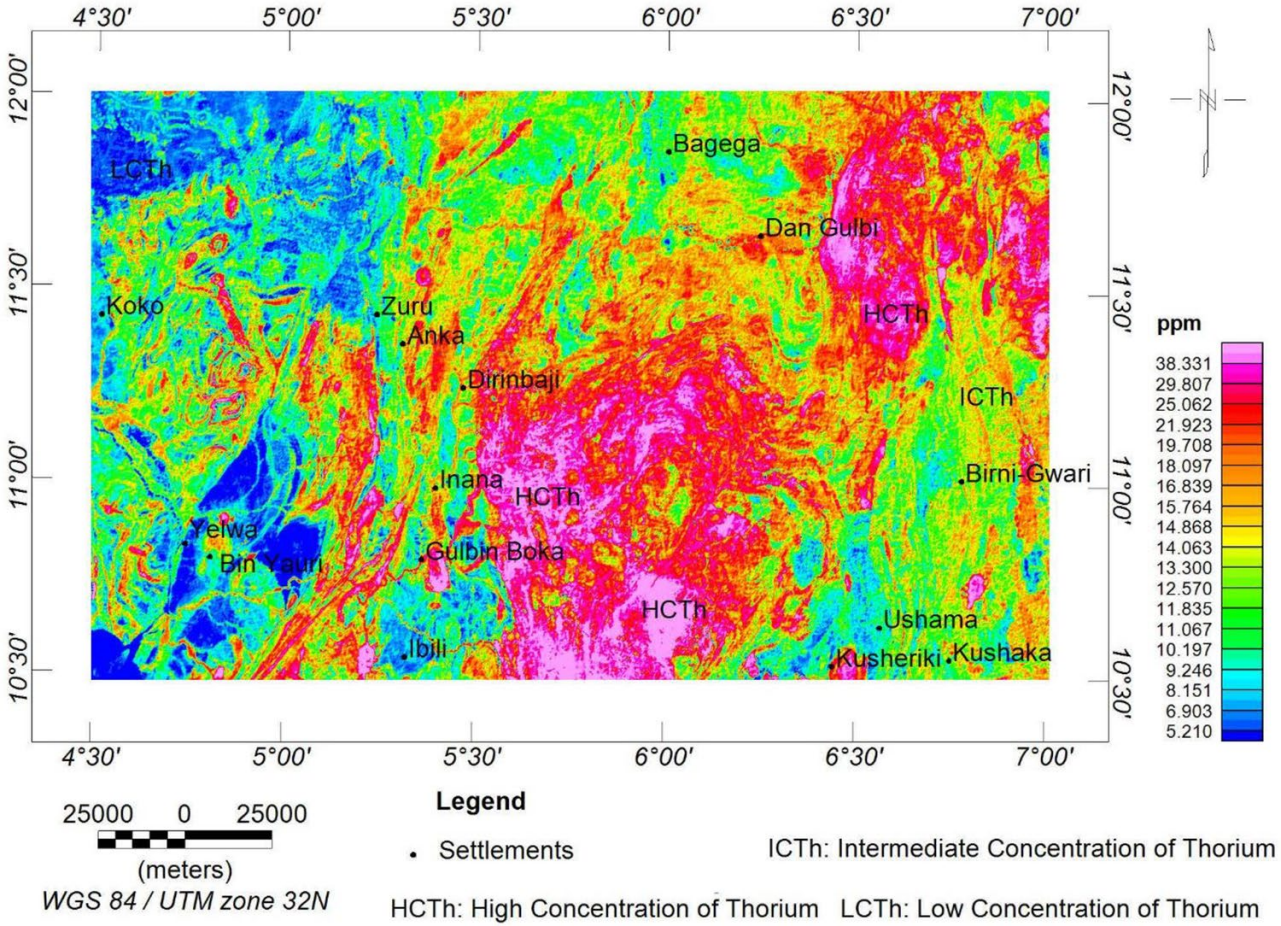
eTh concentration map of the research site.

The equivalent thorium (eTh) map (Fig. 12) exhibits signatures similar to those of the uranium concentration map, which implies a similar lithological classification. However, the high-level concentration of thorium is delineated to be in the range of 18.1 to > 38.3 ppm, while the intermediate and low concentrations are between 11.8 and 18.1 ppm and 5.2 and 11.8 ppm, respectively.

**Figure 13:**
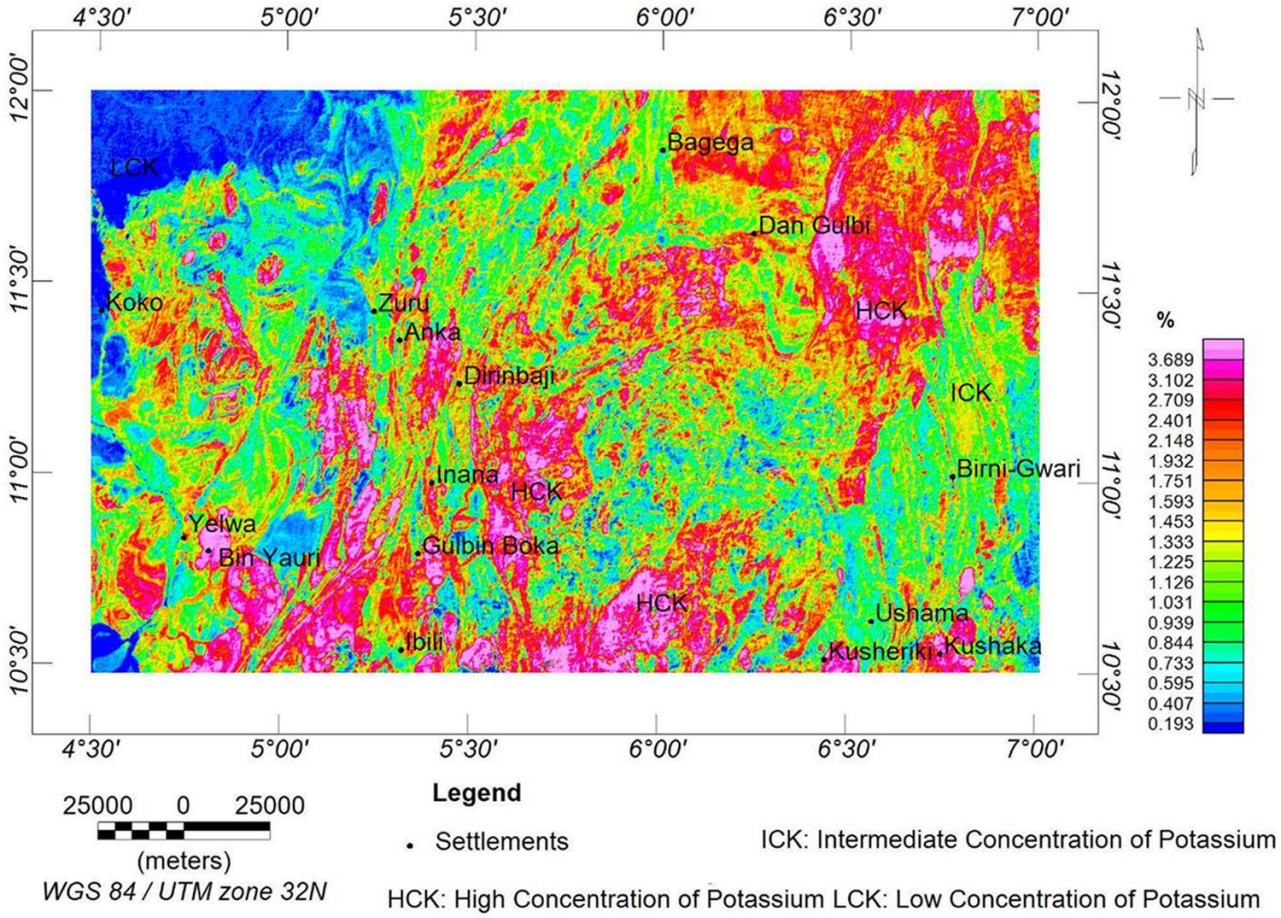
K concentration map of the research site.

The percentage potassium (K) concentration exhibits a similar signature with the equivalent thorium and uranium maps. However, the extent of concentration is depleted (Fig. 13), which may be a result of alteration processes. The higher, intermediate, and lower levels of K concentration are in the ranges of 1.9 to > 3.7%, 0.9 to 1.9%, and < 0.2 to 0.9%, respectively. The higher level depicts the granitic and migmatitic rocks; the intermediate level depicts the schist and phyllites; and the lower level is the claystones, sandstones, pebbles, and grits.

The radioelements (i.e., eU, eTh, and K) exhibit similar concentration levels in various locations. However, the eU and eTh are more identical, owing to the depletion in the K that occurs in the south-central portion of the research area. The depletion could be a result of extensive weathering, and as a result, the potassium content is being decomposed^33^.

#### Ternary map

**Figure 14:**
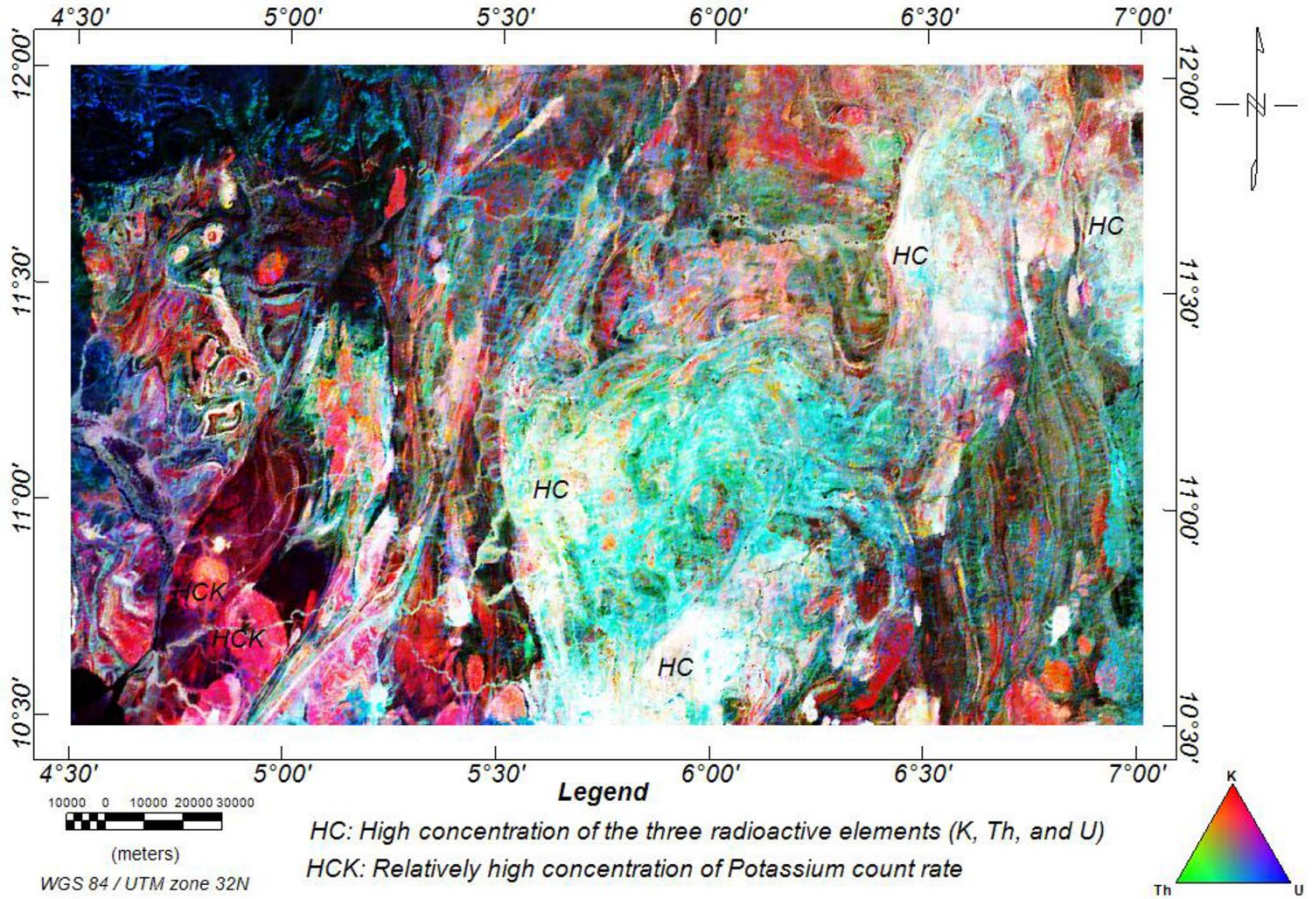
Ternary map of the research site.

Radiometric ternary images are excellent for visualising and enhancing multichannel gamma-ray spectrometry data in a single image^33^. The eU, eTh, and K data were integrated with the colours blue, green, and red, respectively. The white colouration indicates a high concentration of the three radioelements, which are contributions from the porphyritic granite, biotite granite, granite gneiss, and migmatite rocks. In contrast, the black colouration denotes very sparse concentration levels of the radioelements, which correspond to the sedimentary and low-grade metamorphic rocks (Fig. 14). The high concentration zones (HC) are potential targets for radioactive mineralisation^94^. The high concentrations of potassium over thorium and uranium are in red (HCK), which are zones suspected to result from high-level potassic hydrothermal alterations^33^. The green areas are zones of high thorium concentration over potassium and uranium, while the blue colouration is zones of higher uranium concentration over potassium and thorium.

#### K/Th ratio map

**Figure 15:**
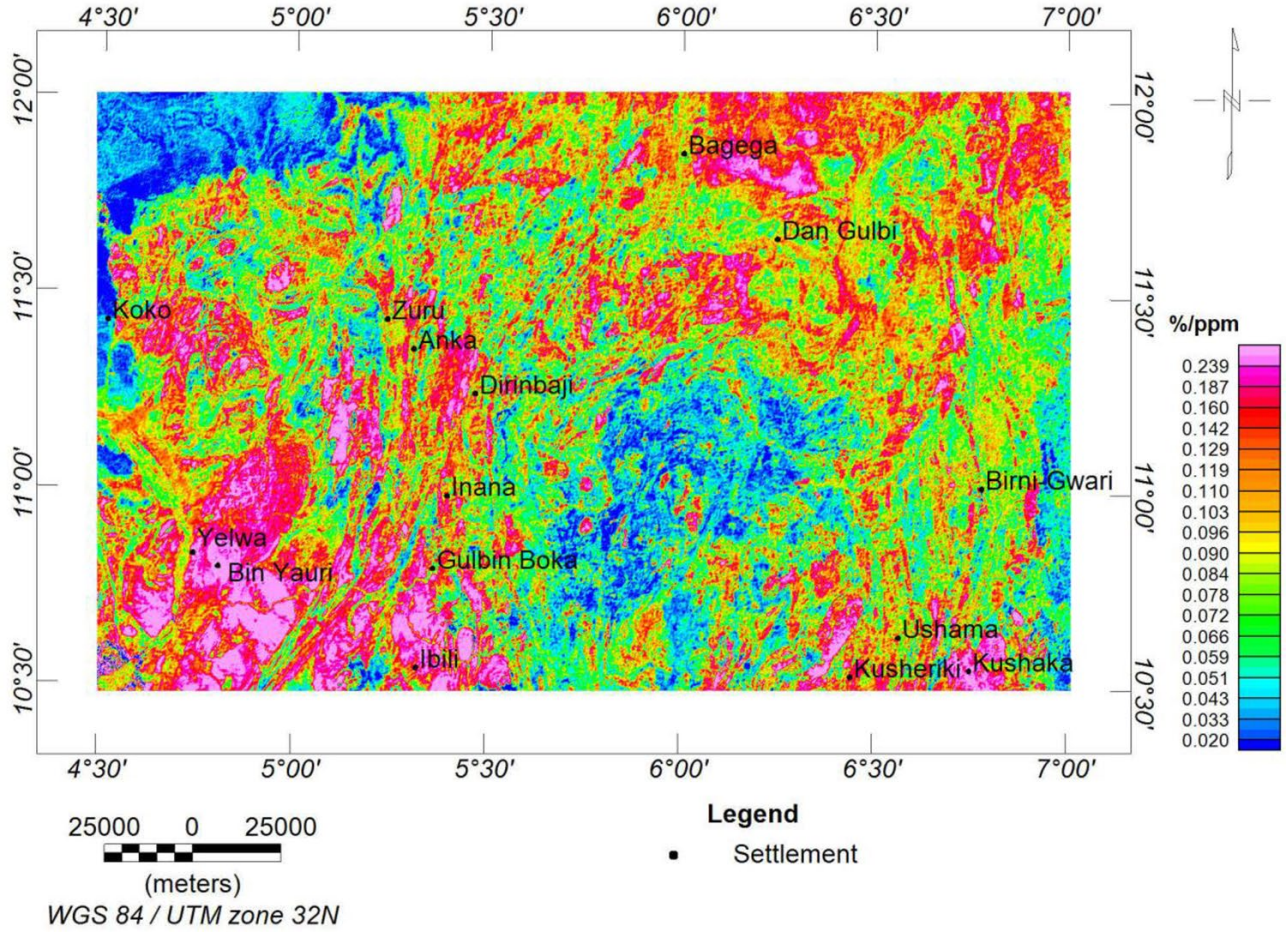
K/eTh ratio map of the research site.

The K/Th ratio is usually effective in delineating hydrothermal alteration zones, and its signatures are usually reliable because they are not usually affected by variations in lithology^18,33,95^. A high level of this ratio could result from high-grade metasomatism at the core of the zones of potassic hydrothermal alterations^96^. This high-level potassic alteration is also observed in the ternary map (Fig. 14). The high K/Th ratio map values indicate potential mineral deposits^20,21^. This study's K/Th ratio map indicates a range of value of 0.020 to 0.239%/ppm. K/Th ratio greater than 0.142%/ppm is considered the hydrothermally altered zone, corresponding to the pink colouration (Fig. 15).

#### Reclassification and spatial interoperability of results

**Figure 16:**
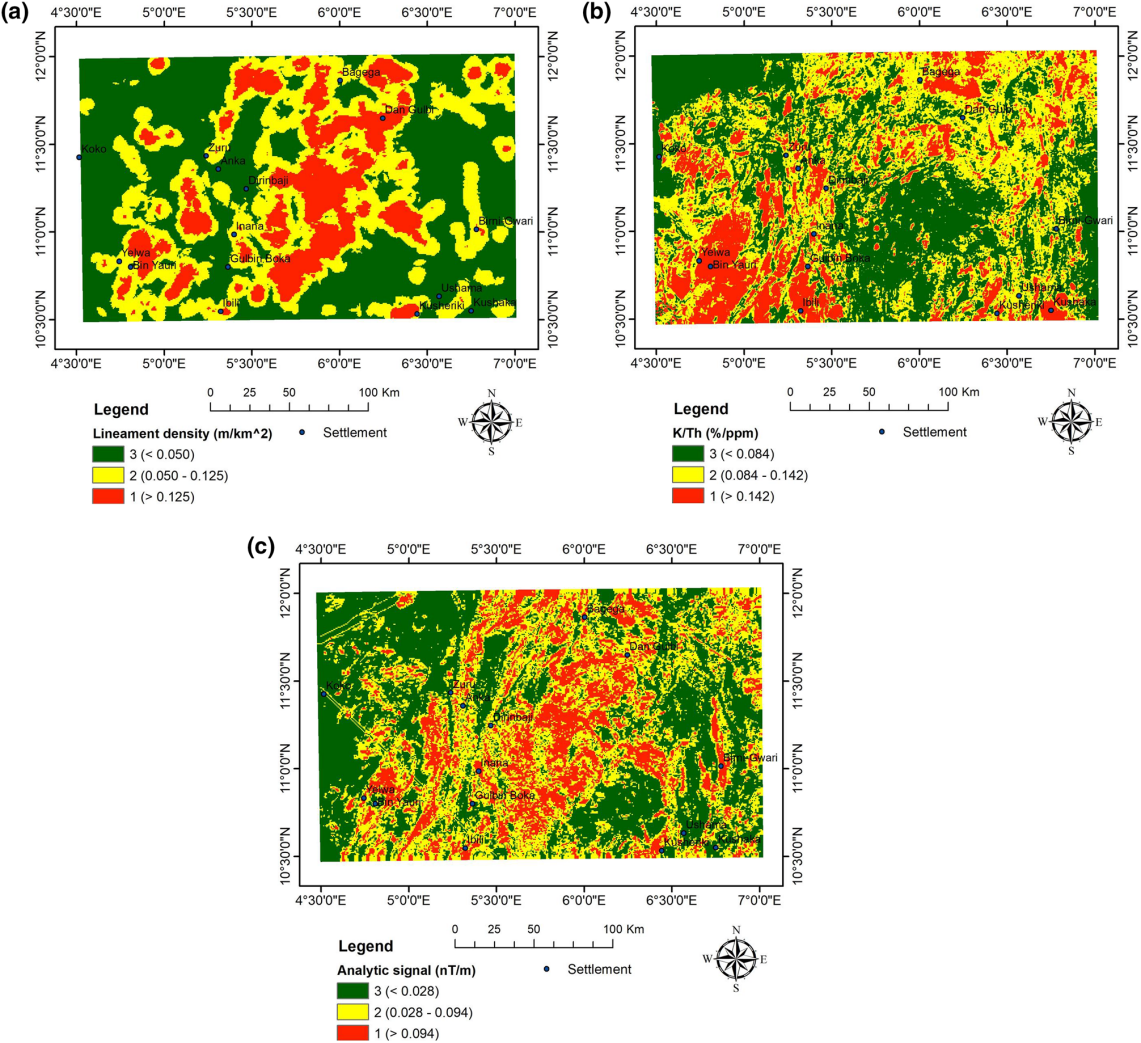
(**a**) Reclassified lineament density map. (**b**) Reclassified K/Th ratio map. (**c**) Reclassified analytic signal map.

The mineralisation potential of northwestern Nigeria is the specific target. The analysis's criteria were adjusted and assigned to three alternate suitability (i.e. mineralisation potential) classifications: Low, moderate, and high potential (Fig. 16a,b,c) (Table 4).

The AHP results are summarised in Table 5. A competent conclusion regarding the importance scale must define the prospect for mineralisation in the area under consideration. Three criteria were selected based on the act and its reliability in determining the mineralisation potential. The analytic signal has the highest cumulative weight index CWI, 0.411. The lineament density and the K/Th ratio are 0.328 and 0.261, respectively. The permissible limit (0.10 or < 10%) set by^77^ is met by the CR of 0.0479 (about 4.79%).

**Table 5:** AHP results for the mineralisation potential.

Pairwise comparison matrix				CWI	Consistency Result
	Lineament density	K/Th	Analytic signal		
Lineament density	1	1	1	0.328	CI = 0.0278 RI = 0.58 CR = 0.0479
K/Th	1	1	½	0.261	
Analytic signal	1	2	1	0.411	

**Figure 17:**
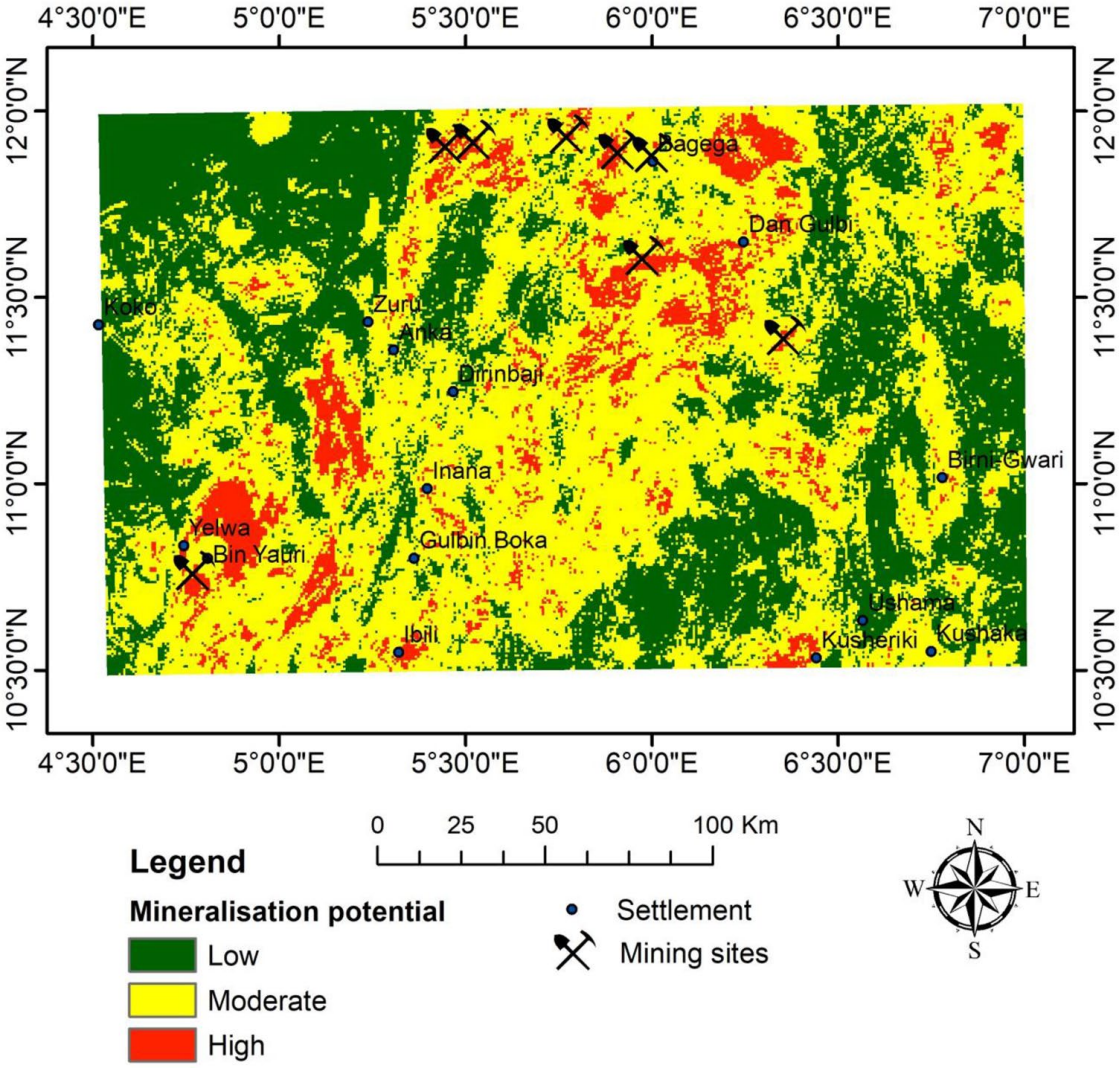
Mineralisation potential map of the study area.

Based on the standard scale and weights assigned, the weighted overlay tool revealed the potential mineralisation of the study area (Fig. 17) using the raster layer (of the analytic signal, lineament density, and K/Th ratio). The mineralisation potential is classified into high, moderate, and low.

#### Validation of results by mines

In order to determine the efficacy of the highlighted target zones for mineralisation potential, validation was performed by posting eight (8) coordinates of active mining sites for correlation. As a result, seven (i.e. 87.5%) of the mine sites fall within the high mineralisation potential. At the same time, 12.5% (i.e. one mining site) fall within the moderate mineralisation potential. This mining site is within the Begega settlement (Fig. 17). None of the mining sites falls under the low mineralisation potential. Therefore, a 100% agreement is met.

## Conclusion and findings

Aeroradiometric and aeromagnetic data were integrated using AHP to reveal the mineralisation potential within northwestern Nigeria. The ASM shows all the edges of anomalous occurrences, structural patterns, and lithological contacts dependent on the magnetisation of different rock compositions. As a result, the site was classified into three magnetolithologic zones: high (> 0.094 nT/m), intermediate (0.028 to 0.094 nT/m), and low magnetic zones (< 0.028 nT/m). The high magnetic zones (HMZ) are the main magnetic source outlines, inferred to be dominantly intrusive zones. The 3-DEuD reveals highly magnetic and intrusive depth sources to be within < 100 to 500 m range. The CET grid technique was used to extract the structures within the research site. The dominant structural trends are E-W, NE-SW, WNW-ESE and NW–SE. The highly dense structural zones coincide with the high magnetic zones and high-frequency amplitudes of the ASM and the FVGM, respectively. Additionally, the CET porphyry detects the centres of the intrusive porphyries to be within zones of high lineament density. This reveals that the mineralisation potential of the area is structurally controlled. The structures are responsible for the hydrothermal mobilisation and concentration of mineralising fluids. A lineament density map was produced to aid the mineralisation potential of the area. On the other hand, the radioelement maps (eU, eTh, and K%) were used for lithological classification, and the radiometric ternary image reveals highly radioactive zones and the superior concentration of individual radioelement at their respective areas. It also reveals some highly potassic alteration zones, which agree with the K/eTh map. The K/eTh ratio map was used to delineate hydrothermal potential zones.

The AHP model was used to integrate the aeromagnetic and aeroradiometric datasets. The AHP result delineates the study site's mineralisation potential into high, moderate, and low. This result was validated using known mine sites. There was a total agreement, with 87.5% of mines plotting within the high mineralisation potential and 12.5% in the moderate class. Other areas of potential mineralisation have been revealed.

## Data Availability

The high-resolution aeromagnetic and aeroradiometric data are not publicly available but can be obtained from the Nigerian Geological Survey Agency. However, both authors can make the data available upon reasonable request.
